# Codification and Prospect of China’s Codification of Environmental Law from the Perspective of Global Environmental Governance

**DOI:** 10.3390/ijerph19169978

**Published:** 2022-08-12

**Authors:** Kai Xu, Guangdong Tian

**Affiliations:** 1 School of International Law, East China University of Political Science and Law, Shanghai 200042, China; 2 Transportation College, Jilin University, Changchun 130012, China

**Keywords:** China, environmental code, international reference, native characteristics

## Abstract

Global environmental governance is the fundamental way to solve the human environmental crisis. With China as the world’s largest emitter of greenhouse gases, the development of China’s environmental law is a key component of global environmental governance. In order to better realize the construction of an ecological civilization, the compilation of China’s Environmental Code has been officially put on the work schedule of the legislature. The compilation of the code is a sincere action, showing that China has taken the initiative to assume its own responsibility for environmental governance. In the past 50 years, China’s environmental legislation has achieved a great leap forward: from nothing to something, from something to something more comprehensive. Aside from this progress, defects such as the internal imbalance of the environmental law system, the backwardness of some environmental legislation ideas, and the inability of environmental legislation and its academic research to fully match China’s national conditions also exist. With the helping hands of conditions and times, it is most appropriate for China to start the compilation of the Environmental Code now. Environmental Codes such as the Swedish Environmental Code, the French Environmental Code and the German Environmental Code (Draft of the Committee of Experts) provide many empirical references for the compilation of China’s Environmental Code. China will make important an contribution to world environmental governance again—an Environmental Code in line with international standards while maintaining native characteristics.

## 1. Introduction

In the 1960s and 1970s, the global environmental crisis awakened people’s awareness of environmental protection, and the global environmental movement has since kicked off [1,2,3]. Human beings have become more and more aware that the environmental crisis is a major global issue, and countries around the world must join hands to effectively alleviate the trend of environmental degradation. The concept of global environmental governance has gradually emerged in the discourse system of international environmental politics [4,5,6,7]. Some scholars have pointed out that there are actually three different ways of using global environmental governance [8]. The global environmental governance referred to in this article is closer to the global environmental governance in the context of international environmental politics, that is, the mechanism for promoting the improvement of the global environment mainly through cooperation between governments and international organizations.

Since the United Nations Conference on the Human Environment in 1972, there has been some substantial progress in global environmental governance. Representative international institutions or organizations on global environmental governance such as the United Nations Environment Programme and the United Nations Intergovernmental Panel on Climate Change have emerged, and a series of conventions and agreements on environmental protection have been formulated, which have made positive contributions to global environmental governance [9]. However, in the past few decades, various environmental crisis events seem to indicate that the environment upon which human beings depend has not been fundamentally improved with the improvement of environmental protection awareness and environmental protection technology. The current global environmental governance system has not been effectively implemented, and global environmental governance still has a long way to go [10]. In the past few years, the development of environmental protection in the world has had its ups and downs. The sudden withdrawal and re-entry of the United States to the Paris Agreement is one of the landmark events of the tortuous development of human environmental protection [11,12]. In July 2022, as one of the pioneer countries supporting the movement for world environmental protection, Germany withdrew the “2035 energy industry greenhouse gas emission neutralization” target in the draft “Renewable Energy Law”, which is the latest example of the setback of global environmental governance [13]. 

Therefore, on the basis of summarizing the experience and lessons of the past 50 years, China proposes that the future global environmental governance should rather advocate a multilateral model based on international law, focusing on fairness and justice, and oriented by effective action, using actual environmental governance actions to replace reliance on international environmental protection conventions [14]. Under such circumstances, in April 2021, the Standing Committee of the National People’s Congress of China formally proposed to start the compilation of administrative and legislative codes, such as the Environmental Code and the education code, where the legislative conditions are ripe [15]. This sends a signal that the codification of China’s Environmental Code no longer stays at the appeal level of scholars and society, but actually enters into China’s national legislative plan. The code is the highest legislative form in the Civil Law System. The compilation of China’s Environmental Code is a major decision to strengthen the legal construction of ecological civilization during the 14th Five-year Plan period of China, and also a symbolic event on the new journey of China to fully build an ecological country. The world’s environmental protection career has suffered twists and turns in recent years.

While as the largest developing country in the world and a country that started late in environmental protection, China has shown great determination in promoting human environmental protection. For example, China’s recently proposed goals of “carbon peaking” in 2030 and “carbon neutralization” in 2060 are particularly showing its energetic environmental strategy [16]. The compilation of China’s Environmental Code is undoubtedly a good embodiment, showing that China is in line with countries advanced in international environmental protection and takes the initiative to assume its own international environmental protection responsibilities. In the context of this new era, the ins and outs of the compilation of China’s environmental code, how it will learn from international experience while maintaining local characteristics, how China uses the environmental code to achieve more effective ecological civilization construction, how to take advantage of the opportunity of compiling the environmental code and how to better promote global environmental governance, are worthy of academic discussion.

At present, the countries that have compiled or tried to compile environmental codes are mainly civil law countries and are of important reference value for China, which is also a civil law system. This article mainly adopts the comparative research method, and discusses the enlightening effect of these environmental codes on the compilation of China’s environmental code through research into the formulation process and characteristics of the Swedish Environmental Code, the French Environmental Code, and the German Environmental Code (Expert Committee Draft).

In recent years, articles introducing the development of China’s environmental law are not uncommon, but relatively few academic articles systematically introduce the compilation of China’s environmental code. Therefore, the main goal of this article is to systematically explain the whole process of China’s environmental code compilation and its possible prospects. This article first discusses the domestic and international situation of China’s environmental governance, which is also an indispensable realistic background for the study of the compilation of China’s environmental code. Next, this article elaborates the development process of China’s environmental law and its shortcomings, and explains the necessity and feasibility of compiling an environmental code in China. Then, this article describes the compiling experience of the Swedish Environmental Code, the French Environmental Code, and the German Environmental Code (drafted by the expert committee) in detail, as well as the theoretical analysis of them by Chinese scholars. Finally, this article gives some suggestions on the compilation mode of China’s environmental code.

## 2. Domestic and International Situation of Environmental Governance in China

Since the reform and opening-up in 1978, China’s achievements in economic development have been recognized by the world, but like most industrialized countries, China’s economic development, especially industrial development, has also caused serious problems [17]. Environmental crises such as air pollution, soil and water pollution, and the reduction of biodiversity have seriously threatened the healthy life of the Chinese people and the sustainable development of China [18]. Therefore, green development has become the only option for China’s future.

### 2.1. Domestic Situation of Environmental Governance in China

It is widely known that, at the expense of ecological and environmental sacrifices, China has achieved rapid economic development in the past few decades [19]. The excessive cost of resources and environment for China’s economic growth has become a major test for China’s economic and social development at present and in the future [20].

In recent years, China has stepped up efforts to control environmental pollution. In particular, China’s legislation on environmental crimes and statutory penalties have been constantly improved, and the number of environmental crimes filed has increased dramatically [21]. Although these measures are a great deterrent to all types of polluters, China’s environmental protection still face multiple challenges. First of all, China’s overall environmental pollution and ecological damage are still relatively serious. Due to its large population, China has the greatest pressure on economic development and employment in the world [22]. China’s long-used extensive economic development mode has been improved to some extent, while its industrial structure has not been fundamentally optimized. The amount of yearly added pollutants is still comparatively high, and some backward production capacity is likely to remain, putting China under the world’s biggest pressure to protect the environment. Secondly, China is also facing a number of emerging environmental crises, especially e-waste and hazardous chemicals, which are further complicating its environment crisis [23]. For example, while China’s long-standing environmental problems such as air and water pollution have not been effectively solved, soil pollution, especially soil pollution caused by heavy metals, has also been exposed, meaning China is always in a state of dealing with environmental crisis. Thirdly, some potential environmental pollution problems continue to emerge. Persistent organic pollutants and other long-term accumulated environmental pollution problems are beginning to be exposed. In autumn and winter, pollution problems such as haze in big cities and urban agglomerations become increasingly prominent [24]. The economic income of the Chinese people has increased significantly with the rapid development of China’s economy. In 2021, China’s per capita GDP had reached USD 12,500, which is quite close to the standard of high-income countries [25]. In this reality, Chinese people are increasingly yearning for a beautiful environment. This aspiration, on the one hand, puts increasing pressure on the Chinese government’s shoulders to protect the environment, while it is also a powerful driving force for China to embark on the path of green development [26].

### 2.2. International Situation of Environmental Governance in China

In environmental governance, China attaches great importance to cooperation with the international community. Since the 1970s, China has been an important participant in promoting international environmental cooperation and facilitating the signing of related treaties. As the world’s largest developing country, China attaches great importance to and is deeply involved in global environmental governance, and has made important contributions to global environmental cooperation in areas such as climate change and biodiversity protection. So far, China has participated in the following major international environmental treaties: the Vienna Convention for the Protection of the Ozone Layer (1985), United Nations Framework Convention on Climate Change (1992), Convention on Biological Diversity (1992), Kyoto Protocol to The United Nations Framework Convention on Climate Change (1997), the Paris Agreement on Climate Change (2016), etc. China is not only a participant in these international environmental conventions, but also an active practitioner, and China is one of the few countries that has fulfilled their targets and tasks on schedule or even ahead of time.

However, in the complex international environmental governance, China has not only won praise, but also constantly faces international pressure that cannot be underestimated [27]. Firstly, international pressure on China to protect the environment is not groundless. As a well-deserved “world factory”, China is the largest carbon emitter globally. In 2019, the world’s total carbon emissions were 10.285 billion tons, while China’s total carbon emissions were 2.777 billion tons, accounting for 27%. The United States accounted for 11%, in second place, and India accounted for 6.6%, in third place [28]. In 2020, China accounted for 30.7% of global carbon emissions [29], while in terms of per capita carbon emissions, China ranks 15th in the world, and is significantly lower than developed countries as a whole [30]. In any case, China faces an extremely tough task to reduce its emissions. Secondly, the rapid development of China’s national strength has broken the international political and economic pattern formed after World War II, which has caused dissatisfaction and suspicion among some countries in the world. In the international arena, hard-line views such as “China is a threat to the climate” and “China should be responsible for the environment” have emerged frequently, and even evolved into political and economic pressure against China in international environmental negotiations [31]. In essence, this is the competition and game of international politics and economics in the new era. Thirdly, with the continuous growth of China’s national strength, China’s international influence is increasing day by day. Moreover, as one of the five permanent members of the UN Security Council, China should also actively assume its responsibilities in environmental protection and establish its image as a major country. As the largest developing country, fulfilling its emission reduction obligations consciously and on schedule and assuming international responsibilities for environmental governance, all of these would help China play its rightful leading role in global environmental governance [32]. On the one hand, it can urge developed countries to honor their emission reduction commitments, and on the other hand can help China work with all developing countries to reject unreasonable emission reduction requests.

All in all, as a responsible major country, China’s domestic and international situation in environmental governance pushes China to make rapid progress towards green and sustainable development.

## 3. The Development Course and Existing Problems of Environmental Law in China

The occurrence of environmental damage and environmental crisis constitute the logical starting point of environmental law [33]. In the history of human development, industrialization has been achieved at a high rate and has incurred environmental crises that seriously endanger people’s lives and health—this is an important reason for the thriving of environmental law in Western countries where environmental protection first developed. In the early days of the founding of the new China, the country’s industrialization was still in its infancy, and the environmental pollution caused by it was not serious. Even so, in the 1950s, China promulgated the Provisional Outline of Water and Soil Conservation and the Trial Regulations on the Protection of Mineral Resources, which both encouraged the exploitation of resources and emphasized the protection of the natural environment [34]. In 1972, China participated in the United Nations Conference on the Human Environment held in Stockholm. Subsequently, China held the first National Conference on Environmental Protection, officially announcing the beginning of China’s environmental protection movement [35]. In the past 50 years, the development of environmental law in China can be roughly divided into the following three stages.

### 3.1. Initial Exploration Period: 1972–1989

In October 1971, China was formally restored to its lawful seat in the United Nations. In June 1972, China participated in the Conference on the Human Environment held in Stockholm, which was the first important international conference China attended after the restoration of its lawful seat. It was also the starting point for China to think about itself and human environment-related issues at a substantive level. Furthermore, it is also the starting point of the initial exploration period of China’s environmental rule of law. In 1973, China held its first National Conference on Environmental Protection, and in 1974 the Central People’s Government established the leading group for environmental protection. Article 11 of China’s 1978 Constitution clearly stipulates that “China protects the environment and natural resources, prevents and controls pollution and other public hazards”. The milestone in the initial exploration of China’s environmental legal system was the Environmental Protection Law (for Trial Implementation) promulgated in 1979. Since then, China has enacted a number of specific laws on environmental protection, such as the Marine Environmental Protection Law (1982), Law of the People’s Republic of China on the Prevention and Control of Water Pollution (1984), Law of the People’s Republic of China on the Prevention and Control of Atmospheric pollution (1987), and Law of the People’s Republic of China on the Protection of Wildlife (1988).

### 3.2. Rapid Development Period: 1989–2012

After laying the foundations in the initial exploratory period and against the background of the rapid development of China’s industry after the reform and opening-up, China’s environmental law entered a period of rapid development, during which there were several events of landmark legislation. First, on the basis of studying the practical effects of the *Environmental Protection Law (for Trial Implementation)*, China amended the Law and promulgated the official version of it in December 1989. Second, in 1992, China participated in the United Nations Conference on Environment and Development held in Brazil, which is widely regarded as an important milestone in the objective of contemporary human environmental protection. At this conference, the concept of sustainable development was recognized and accepted by most countries in the world. In this context, China issued a series of important environmental laws, such as *Law of the People’s Republic of China on Prevention and Control of Environmental Pollution by Solid Waste* (1995), *Law of the People’s Republic of China on Prevention and Control of Environmental Noise Pollution* (1996), and *Law of the People’s Republic of China on Conserving Energy* (1997). Third, since 2000, China has continuously reviewed its environmental protection experience, strictly enforced environmental protection law, and continued to issue important environmental protection laws such as the following: *Law of the People’s Republic of China on Prompting Clean Production* (2002), *Law of the People’s Republic of China on Regenerating Energy Resources* (2005), *Law of the People’s Republic of China on Promoting the Development of a Recycling Economy* (2008), *Law of the People’s Republic of China on the Protection of Offshore Islands* (2009).

### 3.3. Steady and Optimized Period: 2012–Present

Since the 18th National Congress of the Communist Party of China, the development of the environmental rule of law in China has entered a new stage. In 2018, the philosophy of ecological civilization was written into China’s Constitution and has now become an important part of the “Five in One” overall layout of socialism with Chinese characteristics (i.e., economic construction, political construction, cultural construction, social construction and ecological civilization construction). In the new era, China’s environmental protection law has entered a stage of stability and optimization. In 2015, the *Environmental Protection Law* was comprehensively amended. Since then, the *Nuclear Safety Law* (2017), *Soil Pollution Prevention and Control Law of the People’s Republic of China* (2019), *Yangtze River Protection Law* (2021) and other environmental laws have been promulgated.

From the abovementioned, it can be seen that, as a late-developing country in environmental protection, China is catching up and paying increasing attention to environmental protection. Through 50 years of effort, basically all aspects of China’s environmental protection have laws to follow [36]. However, China’s “emergency-like” legislative approach to environmental protection law has accumulated many problems in China’s environmental law. For example, although China has enacted a considerable number of environmental protection laws, there are shortcomings such as uncoordinated legislation, legal conflicts and difficulty in judicial application; and at the level of legislative concept, there is no harmony among the dozens of current environmental laws. Some environmental laws focus on the guarantee of economic development, while others attach more importance to ecological protection. To compound this, China began to compile the Environmental Code.

## 4. The Necessity and Feasibility of Compiling an Environmental Code in China

Since the 1970s, China’s environmental legislation has achieved a great leap forward: from nothing to something, from something to something more comprehensive. However, an environmental law system with advanced concepts, comprehensive function and internal coordination has not been established. Therefore, the Chinese legislature has put the compilation of the Environmental Code on the agenda.

### 4.1. The Necessity of Compiling an Environmental Code in China

First, the internal balance of China’s environmental law system is in urgent need of unification. After decades of development, China has enacted more than 30 laws on environment, environmental pollution and natural resource protection, more than 60 environmental administrative regulations formulated by the State Council, and more than 600 environmental administrative rules formulated by the competent department of the State Council [34]. In such a large and complex legal system of environmental protection, the friction and conflict between laws is inevitable. According to the Water Act in China, for example, extraction of groundwater should be applied to the water resources administrative departments (such as the Water Authority) for water intake permits, while the State Council issued “Interim Measures for Administration of Registration of Mineral Resources Exploration” identifies groundwater as a mineral resource, the use of which requires the application for a license from the relevant administrative department of geology and minerals. Another example is that, China has enacted four water-related laws on environmental protection, namely, the Water Act, the Law of the People’s Republic of China on the Prevention and Control of Water Pollution, the Law of the People’s Republic of China on Water and Soil Conservation and the Flood Control Law, which are, respectively, in the field of economic and administrative law. Although China has a number of environmental protection laws related to water resources, these laws do not form a corresponding legal force, but to some extent cause the waste of legislative resources. Similar problems to these are not uncommon in China’s environmental legal system. Therefore, against the background of China’s in-depth promotion of the rule of law, to improve the actual effect of environmental laws, it is necessary to systematically sort out and unify the environmental laws that have been extensively formed.

Second, China’s environmental law system needs to be governed and standardized by the philosophy of ecological civilization. For a long time, China’s approach to environmental legislation has been like “fire-fighting”. When a certain area of environmental pollution is serious, society and the public react strongly, the relevant environmental laws will be issued quickly. At the same time, since the modern environmental protection movement originated in Western countries, advanced environmental legislation experience and complete environmental law theoretical systems naturally also mainly come from the West. These experiences and knowledge have brought great help to China’s own environmental protection movement and the development of the environmental rule of law, while it also makes China’s environmental legislation and academic research passively restricted within the Western legal framework and theoretical system, unable to fully summarize and reflect China’s own environmental problems. For example, the Chinese environmental law community have long centered on the Western theoretical as “Anthropocentrism” and “Ecocentrism” in environmental legislation [37], and the environmental theories of “the theory on rights of future generations” [38] and “public trust doctrine” [39] are also very popular in Chinese environmental law circles. However, while these theories have greatly inspired China’s environmental law and academic research, time has also proved that China’s social reality and cultural soil cannot fully match with them. Since the concept of ecological civilization was written into the Constitution, China’s environmental rule of law has entered the era of “Constitutional priority”, and the environmental law system, which is subordinate to the Constitution, should accept the unified norms of ecological civilization.

Third, the compilation of the Environmental Code will help China’s environmental protection work to better integrate into and communicate with the international community. At present, the environmental issue is a major realistic issue faced by all mankind. In recent years, Chinese leaders have made their voices heard at many important international conferences and solemnly stated that China is willing to cooperate with other countries and international organizations to promote global ecological and environmental governance [40]. A sound ecological environment is both a natural asset and an economic asset, which has a bearing on the potential and driving force for economic and social development. It should more equitably benefit people of all countries so as to build a home for the common development of all countries [41]. Through the compilation of the Environmental Code, countries around the world can more truly feel China’s determination to carry out its environmental protection strategy and, through the provisions, more clearly understand the status quo and characteristics of the development of China’s environmental rule of law. In other words, the Environmental Code is a concentrated embodiment of the “China proposal” for environmental pollution control, highlighting China’s sincere desire to build a community with a shared future for mankind. Through the compilation of the Environmental Code, countries around the world can better understand China’s stance on environmental protection, and take this as a basis to strengthen communication and exchanges with China on environmental protection, so as to seek the fruits of global ecological civilization construction.

### 4.2. The Feasibility of Compiling an Environmental Code in China

First, China’s compilation of an Environmental Code coincides with a rare domestic and international opportunity. China has entered a new era of socialism with Chinese characteristics since its founding, especially after four decades of rapid development through reform and opening-up. At present, the contradiction between unbalanced and inadequate development and the people’s ever-growing needs for a better life has become the main contradiction in Chinese society. A safe and beautiful natural environment is the foundation of a better life for the people, and its significance is self-evident. Under such circumstances, China has been attaching more importance to environmental protection in recent years. The *Civil Code* of China, which took effect on 1 January 2021, not only inspires the enthusiasm of compiling the Environmental Code, but also provides good experience and reference for the compilation. Therefore, there is a very favorable domestic environment for China to start the compiling work at present. At the same time, the international community has paid more and more attention to environmental protection, and the vast majority of countries have expressed their sincere willingness to cooperate with China in environmental protection. Even the United States, which has had a lot of friction with China in economic and trade fields in recent years, has expressed its clear understanding of the need to cooperate with China on environmental protection issues since the Biden administration took office. China’s Environmental Code will therefore be applauded and supported by the international community.

Second, China’s existing environmental legislation provides a good basis for the codification of an Environmental Code. It is clear from the above that China’s legislation on environmental protection has been growing rapidly since the reform and opening-up, and the numbers of Chinese environmental protection legislation, administrative regulations and rules have reached the hundreds, basically covering all aspects of the country’s environmental protection movement. In terms of pollution prevention and control, China’s representative laws include the following: *Law of the People’s Republic of China on the Prevention and Control of Water Pollution*, *Law of the People’s Republic of China on the Prevention and Control of Atmospheric Pollution*, *Law of the People’s Republic of China on Prevention and Control of Environmental Pollution by Solid Waste*, *Law of the People’s Republic of China on Prevention and Control of Environmental Noise Pollution*, *Law of the People’s Republic of China on Prevention and Control of Radioactive Pollution, Soil Pollution Prevention and Control Law of the People’s Republic of China*, etc. In terms of natural resources protection, China’s representative laws include *The Law of Land Administration of the People’s Republic of China*, *Law of the People’s Republic of China on Water and Soil Conservation*, *Law of the People’s Republic of China on the Coal Industry*, *Law of the People’s Republic of China on the protection of Wildlife*, *Forest Law of the People’s Republic of China*, *Grassland Law of the People’s Republic of China, Yangtze River Protection Law*, etc.; and in line with the latest developments in science and technology, China enacted the *Biosecurity Law of the People’s Republic of China* in 2020. Therefore, the specific legislation of environmental protection in China sufficiently provides a good legislative basis for the compilation of the Environmental Code, and the judicial practice experience of these environmental laws can make the compilation closer to the Chinese reality, and thus more with Chinese characteristics.

Third, China’s Environmental Code is not designed to show itself. It is supported by abundant international experience and academic research results. In fact, in the world, there are many countries that have completed the codification of Environmental Codes, and the codification in China has more than sufficient international experience to which it can refer. For example, as early as 1989, legislation for the Swedish Environmental Code was on the agenda. After the World Conference on Environment and Development in 1992, the Swedish government firmly adopted sustainable development as a national development strategy and officially started the codification work in 1993. The Swiss Environmental Code came into force on 1 January 1999, and has been in force for more than 20 years now, and is regarded as the first Environmental Code of substantial importance to mankind [42]. Another example is that, similar to the time when Sweden started its codification, France published its legislative ideas and work plan for environmental codification through its national environmental plan in 1990, and formed a preliminary draft in 1996. In 2007, all the contents of the French Environmental Code were reviewed and adopted, marking French environmental law being presented in the form of a Code [43]. In addition to successful experiences, the lessons learned from the failure of codifying Environmental Codes in other countries have also brought inspiration and benefit to China’s codification. For example, Germany, as one of the most technically advanced countries in the civil law system and attaching the greatest importance to environmental protection, proposed the legislative concept of an Environmental Code as early as the 1970s and even produced four high-quality legislative drafts—the “Professorial Draft” (1994), the “Expert Committee Draft” (1997), the “Working Draft” (1999), and the “Legislative Draft” (2009). However, the legislation of the German Environmental Code ended in failure due to federal and local competition and mutual constraints of different government departments. In a word, the compilation of China’s Environmental Code has sufficient international experience and lessons to reference, making compilation work completely feasible.

## 5. Main International References for China to Compile Environmental Code

***The Swedish Environmental Code***: the Swedish Environmental Code is a good paradigm of a process-led model.

The first Swedish law on forestry was enacted in 1903 and subsequently passed environmental protection laws such as the Water Act, the Hunting Act and the Fisheries Act [44]. After the Second World War, Sweden’s heavy industries such as automobiles, machinery and steel developed rapidly, but the problems of environmental pollution were also becoming more and more serious. As a result, Sweden enacted the Environmental Protection Act in 1969, followed by the Waste Collection and Disposal Act (1979), the Environmental Damage Compensation Act (1986), and the Natural Resources Act (1987) and other important environmental laws. After the Rio Conference in 1992, the concept of sustainable development was officially accepted. Sweden has set the goal of its environmental policy as: Sweden should become a world leader in efforts to create ecologically sustainable development [45]. In order to promote the implementation of sustainable development strategy, Sweden began to compile the “Environmental Code” in 1993. In terms of codification mode, Sweden adopts a substantive codification mode, and 15 important environmental laws are integrated into the environmental code, such as the Environmental Protection Act, the Natural Resources Act, the Nature Conservancy Act, the Health Protection Act, the Water Act, the Agricultural Land Management Act, the Public Cleansing Act and the Environmental Damage Act, etc., [44].

The Swedish Environmental Code is organized according to the environmental governance process of “prevention, regulation and remedy” [46]. The Swedish Environmental Code consists of seven titles, and the general provisions and subprovisions are legislated separately. Titles 1–3 (General Provisions; Nature Protection; Special Provisions for Specific Activities) of the Swedish Environmental Code mainly state the legislative purpose and boundaries of the Code, as well as the rights and obligations of the supervising and supervised subject. Titles 4–6 (Examination of Cases and Matters; Supervision; Penalties) mainly set out the specific ways of environmental regulation in Sweden, and Title 7 (Compensation) mainly sets out the remedies for environmental pollution damage. In addition to being an example of a legislative model, the Swedish Environmental Code is particularly praiseworthy for its pragmatic spirit. Sweden enacted its *Environmental Protection Act* in 1969, but the results were not as good as they could have been. Therefore, Sweden took a very practical approach to the Environmental Code, not aiming for a logically rigorous and perfect one, but rather for a formal code that is largely open to the needs of the country’s social development and implementation capabilities [47]. This legislative model has enabled the Swedish Environmental Code to unify Sweden’s previous environmental law system in a relatively short period of time, and has opened up a new dimension of environmental rule of law in Sweden, providing a lot of successful experiences for China’s Environmental Code codification [48].

What is more worthy of appreciation is that in order to realize the environmental strategy of sustainable development and ensure the implementation of the environmental code and other environmental protection laws, Sweden built a complete environmental court system to make the trial of environmental cases more professional [49]. Although, some changes in Sweden’s environmental policy in recent years have caused some opinion to question and reflect on its status as a leading country in environmental protection and Sweden’s national environmental protection goals [50,51]. However, Sweden is still one of the most successful countries in terms of environmental protection in the world, and the compilation mode of its environmental code and advanced environmental protection ideology are very worthy of reference for China.

***French Environmental Code****: the French Environmental Code* is an example of a factor-led model.

Throughout the legislative history of countries belonging to the civil law system, France undoubtedly has a long tradition of codification, and the *French Civil Code*, which has great significance in history, is still praised by the whole world. However, France’s environmental protection efforts are not ahead of other European countries and have been criticized by social media [52]. It was not until 1971 that a dedicated environment ministry was established for the first time in the French government cabinet. Regrettably, the French Ministry of the Environment at that time only had the function of coordinating and persuading, lacking any management or command power [53]. With the awakening of the French people’s environmental awareness, the French government is paying more and more attention to environmental protection. After the 1980s, environmental rights have been regarded as a basic human right and have become one of the important concepts in the development strategy of the country [54].

*In this context, the French Environmental Code*, which was officially launched in the late 1980s, concentrates on the protection of environmental elements [55]. The code adopts the internal structure of “Volume-Title-Chapter-Section-Sub-section-Article”, and it is worth mentioning that, compared with other countries, the *French Environmental Code* is very large in scale. In addition to the general provisions in Volume 1, the contents of the other six volumes are mostly focused on the protection of different environmental elements, and for different environmental elements, there are different regulatory systems and legal responsibilities to match [56]. The main goal of the French environmental code is to solve a series of problems faced by French environmental law at the end of the 20th century. For example, environmental laws continue to increase, but their content has problems such as having ambiguous, incomprehensible, duplicated and even conflicting regulations. Accompanying it is the governance problem of the repeated establishment of administrative organs and low administrative efficiency. Through the codification work, the French environmental law norms have been incorporated into a unified system, and the basic principles, concepts, systems and procedures of environmental law have been reorganized, simplified and clarified, thus becoming simpler and easier for the public to understand [57].

An important inspiration for China to take from the compilation of the French Environmental Code is that it is very significant to choose and determine the mode of codification at the beginning of the work. At present, there are two main modes of codification in the world: one is formal codification, which systematically integrates existing laws without making substantial changes to them; the other way is to make substantial changes and innovations. Unlike Germany, Sweden and other countries that tried substantive codification, from the beginning of the codification work in France, the formal codification mode (codification à droit constant) has been chosen. Therefore, at the beginning of the codification work, it is necessary to clarify the mode to be adopted and clarify the scope, content and principles of the codification, which can greatly promote the smooth progress of the codification work.

In summary, the *French Environmental Code* took more than ten years to compile, and in the process, it fully demonstrated the democratic principles of modern legislation and actively involved public opinion. This has not only improved the relevance of the content of the code, but also greatly improved the dissemination of modern environmental concepts, so that the code has been generally accepted by society and the public [58].

***German Environmental Code (Expert Committee Draft)***: the code is an example of a mixed structure model.

Among the civil law countries, Germany has a notable tradition of codification, and the German Federal Government and academia have devoted a great deal of effort to the codification of the Environmental Code. However, after two environmental codification campaigns and four different drafts, the *German Environmental Code* did not really come into existence [59]. While these drafts, especially the draft of the *German Environmental Code (Expert Committee Draft)* (hereinafter referred to as “*German Environmental Code*”), is of high legislative level and quality, and is worthy of study and reference by the Chinese legislature and academia. The *German Environmental Code* adopts a more conventional general substructure, with eight chapters in the general provision part, which present the thinking process of the administrative control of environmental pollution as a whole, and nine chapters in the subprovision part, which mainly focus on the protection of different environmental elements. Therefore, the *German Environmental Code* can be said to adopt a mixed structure. In addition to the *German Environmental Code*, the *Italian Environmental Code*, which is not yet completed, also adopts this structure, taking into account the phased characteristics of environmental governance and the differentiated treatment of different environmental elements. Needless to say, in addition to the legislative techniques and structure, the lessons from the failure to compile the *German Environmental Code* are most worthy of reference for China and other countries trying to compile an Environmental Code. On the first try, the codification was aborted due to the conflicts of interests between different government departments. After the former German Chancellor, Angela Merkel, came to power, the drafting of the *German Environmental Code* was at a faster pace, but it was aborted again, due to the resistance of Bavaria, which was regrettable [60]. It can be seen from this, the enactment of an Environmental Code or even any important law requires favorable political, social and international conditions.

## 6. Conclusions

The codification of China’s Environmental Code is in full swing. According to the experience of overseas codification, the work is expected to last for several years to ensure the quality of the code. At present, the Chinese legislature, environmental law academia and the public are still discussing the legislative positioning, structure and content of the Chinese Environmental Code, and the differences on certain issues are still obvious. Now, it seems that the main points that could reach a preliminary consensus on the codification of China’s Environmental Code are the codification mode and legislative structure.

As previously mentioned, in terms of the mode of codification, there has always been a distinction between substantive codification mode and formal codification mode. The former means that the codification should follow the legislative logic to a high degree, with the rational perfection of the structure of the code as the goal, and the exquisite richness of the content and the stability of the code as a pursuit over a longer period of time; the latter means that the work of codification is obtained more efficiently and maintains a better openness of the code, mainly through the mode of legal compilation [61]. Chinese legislators and scholars think that, although substantive compilation can better demonstrate China’s commitment to environmental protection and environmental governance, the conditions are not ripe at the present stage. In reality, the formal codification mode is more suitable for the current environmental codification in China, and “moderate codification” is the inevitable choice [62]. In other words, China’s current social situation is still changing with each passing day, and the reality of environmental pollution in China and the demand for environmental governance are also in a volatile stage. Irrationally pursuing the Environmental Code to be completed in one move may lead to difficulties in the implementation. Therefore, the compilation of China’s Environmental Code should follow the path from environmental specific law to formal code and then to substantive code [63].

With regard to the structural arrangements of China’s Environmental Code, the view of adopting the general substructure has been widely accepted [64]. First of all, in view of the existing Environmental Codes around the world, the method of codification by using the general subprovision structure has been widely used due to its advantages of clear separation of legislative logic and functional boundaries. In the general provisions of the Environmental Code, it usually has macroscopic contents such as the overall and basic purpose, concept and principle of the code, while in the subprovisions of the Environmental Code, the specific institution, division of rights and obligations and relief methods of environmental governance can be specified [65]. Secondly, the adoption of the general substructure is also consistent with China’s legislative habits, as many important branch laws in China adopt it, such as the *Criminal Law*, the *Administrative Permission Law of the People’s Republic of China*, and the *Administrative Punishment Law of the People’s Republic of China*. As the cornerstone of China’s environmental legal system, the *Environmental Protection Law of the People’s Republic of China* also adopts a general substructure. In essence, the general substructure is the legislative structure most familiar to the Chinese legislature and the public, and there is a good reason for the codification of the Chinese Environmental Code to maintain local characteristics. Thirdly, after the structure is determined, the compilation of China’s Environmental Code should also take into account the thinking process and the protection element. Under the guidance of the logic of the environmental pollution control sequence, different institutional arrangements should be made according to the prevention characteristics of the different elements, and at the same time, attention should be paid to ensure that the Environmental Code has a certain openness [66].

The process of compiling China’s Environmental Code has just begun. No matter what legislative system or content it will adopt, it always will be a good demonstration of China’s commitment to global environmental governance and the best representation of China’s efforts to build a community with a shared future for mankind in the environmental field. The compilation work needs to draw on mature international experience and strengthen communication with the international community. Only in this way, can China contribute more Chinese solutions with Chinese characteristics and wisdom to global environmental governance.

## Data Availability

Not applicable.
